# Under-specification as the source of ambiguity and vagueness in narrative phenotype algorithm definitions

**DOI:** 10.1186/s12911-022-01759-z

**Published:** 2022-01-28

**Authors:** Jingzhi Yu, Jennifer A. Pacheco, Anika S. Ghosh, Yuan Luo, Chunhua Weng, Ning Shang, Barbara Benoit, David S. Carrell, Robert J. Carroll, Ozan Dikilitas, Robert R. Freimuth, Vivian S. Gainer, Hakon Hakonarson, George Hripcsak, Iftikhar J. Kullo, Frank Mentch, Shawn N. Murphy, Peggy L. Peissig, Andrea H. Ramirez, Nephi Walton, Wei-Qi Wei, Luke V. Rasmussen

**Affiliations:** 1 Center for Health Information Partnerships (CHIP), Northwestern University Feinberg School of Medicine, 625 N. Michigan Ave, Suite. 1500, Chicago, IL 60611 USA; 2 Northwestern University Feinberg School of Medicine, Chicago, IL USA; 3 Department of Biomedical Informatics, Columbia University, New York, NY USA; 4 Research IS and Computing, Massachusetts General Hospital Brigham, Somerville, MA USA; 5 Kaiser Permanente Washington Health Research Institute, Seattle, WA USA; 6 Department of Biomedical Informatics, Vanderbilt University Medical Center, Nashville, TN USA; 7 Department of Cardiovascular Medicine, Mayo Clinic, Rochester, MN USA; 8 Department of Health Sciences Research, Mayo Clinic, Rochester, MN USA; 9 Center for Applied Genomics, Children’s Hospital of Philadelphia, Philadelphia, PA USA; 10 Biomedical Informatics Research Center, Marshfield Clinic Research Institute, Marshfield, WI USA; 11 Intermountain Precision Genomics, Intermountain Healthcare, St. George, UT USA; 12 Department of Preventive Medicine, Northwestern University Feinberg School of Medicine, Chicago, IL USA

**Keywords:** Electronic Health Records (EHR), Phenotyping, Ambiguity, Vagueness, Under-Specification, Algorithm: Natural Language

## Abstract

**Introduction:**

Currently, one of the commonly used methods for disseminating electronic health record (EHR)-based phenotype algorithms is providing a narrative description of the algorithm logic, often accompanied by flowcharts. A challenge with this mode of dissemination is the potential for under-specification in the algorithm definition, which leads to ambiguity and vagueness.

**Methods:**

This study examines incidents of under-specification that occurred during the implementation of 34 narrative phenotyping algorithms in the electronic Medical Record and Genomics (eMERGE) network. We reviewed the online communication history between algorithm developers and implementers within the Phenotype Knowledge Base (PheKB) platform, where questions could be raised and answered regarding the intended implementation of a phenotype algorithm.

**Results:**

We developed a taxonomy of under-specification categories via an iterative review process between two groups of annotators. Under-specifications that lead to ambiguity and vagueness were consistently found across narrative phenotype algorithms developed by all involved eMERGE sites.

**Discussion and conclusion:**

Our findings highlight that under-specification is an impediment to the accuracy and efficiency of the implementation of current narrative phenotyping algorithms, and we propose approaches for mitigating these issues and improved methods for disseminating EHR phenotyping algorithms.

**Supplementary Information:**

The online version contains supplementary material available at 10.1186/s12911-022-01759-z.

## Background

The process of identifying patients exhibiting a particular phenotypic trait using data from the electronic health record (EHR) has increased the capability of health and biomedical researchers to conduct studies using retrospective data. The process of developing, executing, and disseminating the logic to identify the cohorts and attributes of interest has been referred to as EHR-based phenotyping [1, 2].

The field of EHR-based phenotyping has expanded in the past decade, with progress led by multiple groups such as the electronic Medical Record and Genomics (eMERGE) Network [3, 4], The Patient-Centered Outcomes Research Network (PCORnet) [5], the Informatics for Integrating Biology & the Bedside (i2b2) community [6], and the Observational Health Data Sciences and Informatics (OHDSI) consortium [7]. Earlier phenotype algorithms were primarily rule-based and created through expert curation by multi-disciplinary teams that included clinicians, researchers, informaticians, and data analysts. Within eMERGE, a phenotype algorithm is typically developed by one institution and implemented and validated by at least one other institution for evaluation and tuning to enhance portability before wider release. Historically these phenotype algorithms have been represented as narrative descriptions of the logic, which each institution would then translate into executable code to query a local data warehouse.

The use of a narrative phenotype definition has both pros and cons [8]. Natural language can be extremely flexible and succinct and is a convenient representation for broad dissemination; unlike specific programming language implementations that may require specialized knowledge to interpret. However, free-form natural language is prone to issues of vagueness and ambiguity resulting from under-specification. Here we consider under-specification to be a more general issue in which the lack of sufficient detail and contextual information impedes clear interpretation, resulting in idiosyncratic implementation. Ambiguity, where a statement can be interpreted in multiple legitimate ways, can exist in under-specified criteria. For instances, asking for “patients that are 40 years of age or older” does not indicate at what point in time the patient should be at least 40. Whereas, vagueness, in which a specific term has fuzzy boundaries for a reader, can also happen if a term is under-specified. Examples of this would include the terms “tall” or “young”, which lacks a precise quantitative range, as well as “continuous enrollment” which lacks a single definition applicable to diverse healthcare settings. These issues can compromise the accuracy or consistency of algorithms as interpreted by multiple individuals.

Ambiguity and vagueness have been studied in clinical practice guidelines (CPGs) and phenotype algorithms. For CPGs, a proposed model based upon a literature review accounted for classification of ambiguity and vagueness specifically in CPG recommendations [9]. This model was built on established linguistic definitions of ambiguity, vagueness, and under-specification, but was focused on language commonly used in CPGs, which differs from the language used in a phenotype algorithm. The closest relevant work is the formulation of the biomedical query mediation (BQM) process [10]. In BQM, a data analyst works collaboratively with a medical researcher to take information and data needs (e.g., identifying a cohort of patients and extracting data for analyses) and translate them into executable code to query a data warehouse. In the formulation of the BQM model, the researchers studied both written and verbal communications, and identified several key steps where clarification was needed and sought by the data analyst [11]. In the eMERGE network, there is a similar process for phenotype implementation, where a specification of an algorithm is presented, attempts are made to implement it, and dialog ensues to seek clarification when questions arise. A key difference is that with phenotype implementation across institutions, communication primarily occurs between data analysts, as opposed to data analysts and medical researchers.

Extending the BQM process analysis work [10] to further our understanding of under-specification, ambiguity, and vagueness in narrative phenotype algorithms, we investigated the communication involving the implementation of a collection of narrative phenotype algorithms developed by the eMERGE Network.

## Methods

### Data source

We selected narrative phenotype algorithms developed by the eMERGE Network that were contributed to the Phenotype Knowledgebase (PheKB) [12] and had a status of “Final” (publicly available), “Validated” (implementation and review completed by one site other than the authoring institution), or “Testing” (under evaluation by one site other than the authoring institution). We chose this collection in part because of the authors’ familiarity with their development, but also because the algorithms were developed with the intent of being shared across institutions. PheKB supports discussion threads for each phenotype. Although this may be used at any phase in the phenotype development process (development, validation, implementation), within eMERGE the discussion threads were used after the initial site had developed the phenotype and was making it available for other sites to implement. The discussion threads were where implementers would ask clarifying questions, and as such has a history of collaborative discussion during their implementation. Our focus was on interpersonal communication where questions were identified, and so we considered our data source as the record of discussions between a phenotype author and any of several phenotype implementers.

**Fig. 1:**
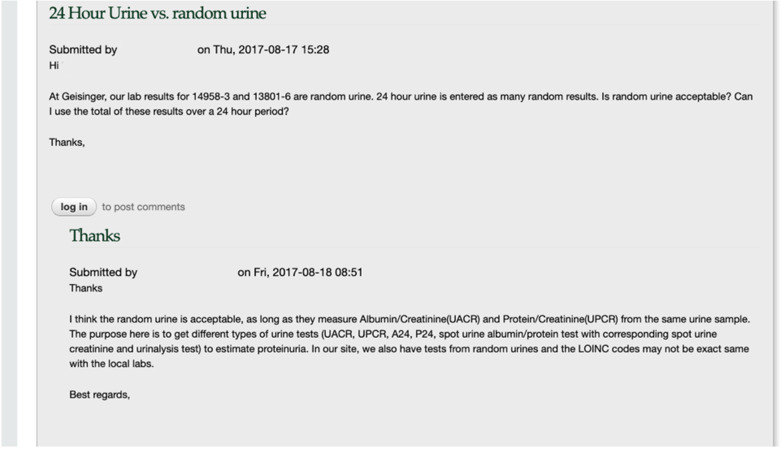
Example of raising issues of vagueness and under-specification in the PheKB database, from the Chronic Kidney Disease phenotype. https://phekb.org/phenotype/chronic-kidney-disease

Although eMERGE members were encouraged to use the PheKB website to discuss questions about phenotype algorithms, email, telephone, and in-person communications were also used. Given that some phenotypes were developed starting in 2007 (when eMERGE phase I began) and that individuals working on phenotypes may no longer be with an institution (in addition to other logistic issues), we deemed it infeasible and therefore out of scope to request and analyze email communication as part of this study. We limited our analysis instead to the written interactions between institutions documented in PheKB’s online discussion forum. An example of these inquiries and comments is shown in Fig. 1. All phenotype comments from PheKB were exported to an Excel spreadsheet.

### Codebook development

Initially, two authors (JY, ASG) independently annotated each PheKB comment for phenotypes having a status of “Final” with descriptive labels for attributes of under-specification. Under-specifications with similar characteristics were categorized together. For instance, under-specifications related to temporal attributes of the phenotype were labeled as “temporal under-specification”. Sub-categories were devised after the overall categories were established. Following the initial annotation process, the two authors consolidated similar labels into a single category and assigned a more comprehensive label. These labels were expanded into a preliminary codebook, including the reconciled labels, detailed descriptions of each category, and examples. The codebook was then reviewed by two other authors (LVR, JAP), who adjusted the category name and descriptions to improve comprehensibility.

### Coding

A second set of phenotypes was selected from PheKB, which included all eMERGE phenotypes having a status of “Validated” or “Testing”. These phenotypes were sufficiently developed to have captured ample user feedback at the time of review. Two groups of annotators were organized to independently annotate the inquiries and comments. The first group included 12 authors from 7 sites (Children’s Hospital of Philadelphia, Columbia University, Geisinger, Harvard University, Marshfield Clinic, Mayo Clinic, and Vanderbilt University Medical Center), and the second group was composed of 3 authors from Northwestern University, who participated in the initial codebook development. Annotators were assigned phenotypes that were not developed or validated by their institution to prevent potential bias. Annotators were provided with the initial codebook and asked to label the user inquiries with the specific category of under-specification found in their assigned phenotypes. We did not consider codes to be mutually exclusive, and coders were instructed to apply all codes they felt were relevant. Annotation was conducted independently and compiled into a spreadsheet to facilitate code reconciliation.

During the coding process, annotators were also asked to provide feedback regarding the codebook, such as requesting clarifications on existing codes or proposals for new codes. The main site team discussed the resulting feedback and made pertinent adjustments to the codebook. The updated codebook was then distributed to the annotators who were asked to reassess their annotations using the new codebook. Finally, each annotator pair reviewed discordant codes in order to arrive at a consensus decision. Feedback from this process was used to further clarify the codebook. The final codebook became our taxonomy for under-specifications and other common errors in the narrative phenotype algorithms.

### Descriptive statistics

We calculated descriptive statistics regarding the prevalence of each under-specification code applied from the reconciled data set using R version 3.6.3 (The R Foundation).

## Results

We extracted the written questions and answers for a total of 34 phenotypes from PheKB, which included 664 messages. The list of phenotypes reviewed are shown in the supplemental Table 1. Of these, 14 phenotypes had a status of “Final”, which included 183 comments. Of those comments, 129 (70%) were found to contain requests for clarification and were ultimately used in the initial development of the codebook.

**Table 1:** Counts of vagueness and under-specification in narrative phenotype algorithms

Code	Category	Sub-category	Description	Total instances	Phenotype count (%)
1.1	Definition of variable	Attributes of variable	Under-specification in attributes (min, max, etc.) of a variable	47	13 (68.4%)
1.1.1.a	Time point	Temporal entity	Under-specification of the time anchor or point of reference for a certain criterion	22	11 (57.9%)
1.1.1.b	Time point	Temporal interval	Under-specification of the range of time you are looking at to find a certain criteria (diagnosis, medication, lab, etc.)	6	5 (26.3%)
1.1.2.a	Threshold	Missing threshold	Vagueness or under-specification for a criterion in the phenotype algorithm	2	2 (10.5%)
1.1.2.b	Threshold	Quantifying qualitative terms	Vagueness or under-specification in the qualitative term describing a criterion (e.g., chronic, young, old, severe, negative, positive) and lacking quantitative values	1	1 (5.3%)
1.1.2.c	Threshold	Units	The units associated with the numeric value (e.g., mg/dL) are not specified	2	1 (5.3%)
1.2	Definition of variable	Alternatives to missing data	Request for instructions when data elements not available	6	5 (26.3%)
1.3	Definition of variable	Code/acronym/term definition	Under-specification regarding acronyms, variables or codes. This could be related to: 1. Local and unique codes 2. Coding/terminology system (including use of base codes) 3. Vague terminology/codes	28	11 (57.9%)
1.4	Definition of variable	Location in EHR	Under-specification regarding how or where certain criteria/variables should be obtained within the EHR	10	6 (31.6%)
2.1	Data dictionary	Data delivery	Under-specification regarding how the data dictionaries should be structured and how to be delivered to site	3	2 (10.5%)
2.2	Data dictionary	Information inclusion	Under-specification regarding what results should be included in the data dictionary	31	10 (52.6%)
2.3	Data dictionary	Results presentation and formatting	Under-specification regarding the formatting of the results in the data dictionary. This may include numeric formatting (e.g., number of decimal places), or granularity of units (e.g., date of birth vs. age)	27	8 (42.1%)
3.1	Logic	Discordant logic	Discrepancy between the written description and the flow chart or the procedures in the flowchart	17	8 (42.1%)
3.2	Logic	Missing rationale or context	Under-specification in the rationale and/or context of the phenotype for its appropriate application	11	8 (42.1%)
3.3	Logic	Population criteria	Vagueness and under-specification in the criteria differences between the case and control or other cohort definitions	20	11 (57.9%)

A total of 304 instances were found across 253 comments (a single comment could exhibit more than one category). Sub-codes are more specific and considered distinct from a higher-level code. Total instances denote the aggregate count of unique instances of under-specifications found across all phenotypes

Dual coding was performed on the remaining 20 phenotypes, which had a total of 481 comments. Of these comments, 253 (53%) comments were found to contain requests for clarification due to under-specification in the phenotype algorithm specification. Since a single comment could exhibit more than one category of under-specification, a total of 304 vagueness and ambiguity instances are present across these 253 comments.

Some of the most common feedback provided during the coding process included queries about nomenclature and requests for further definition of borderline cases. The codebook was revised after the annotators finished the first round of coding and continued iteratively as the two groups of annotators completed reconciliation.

**Fig. 2:**
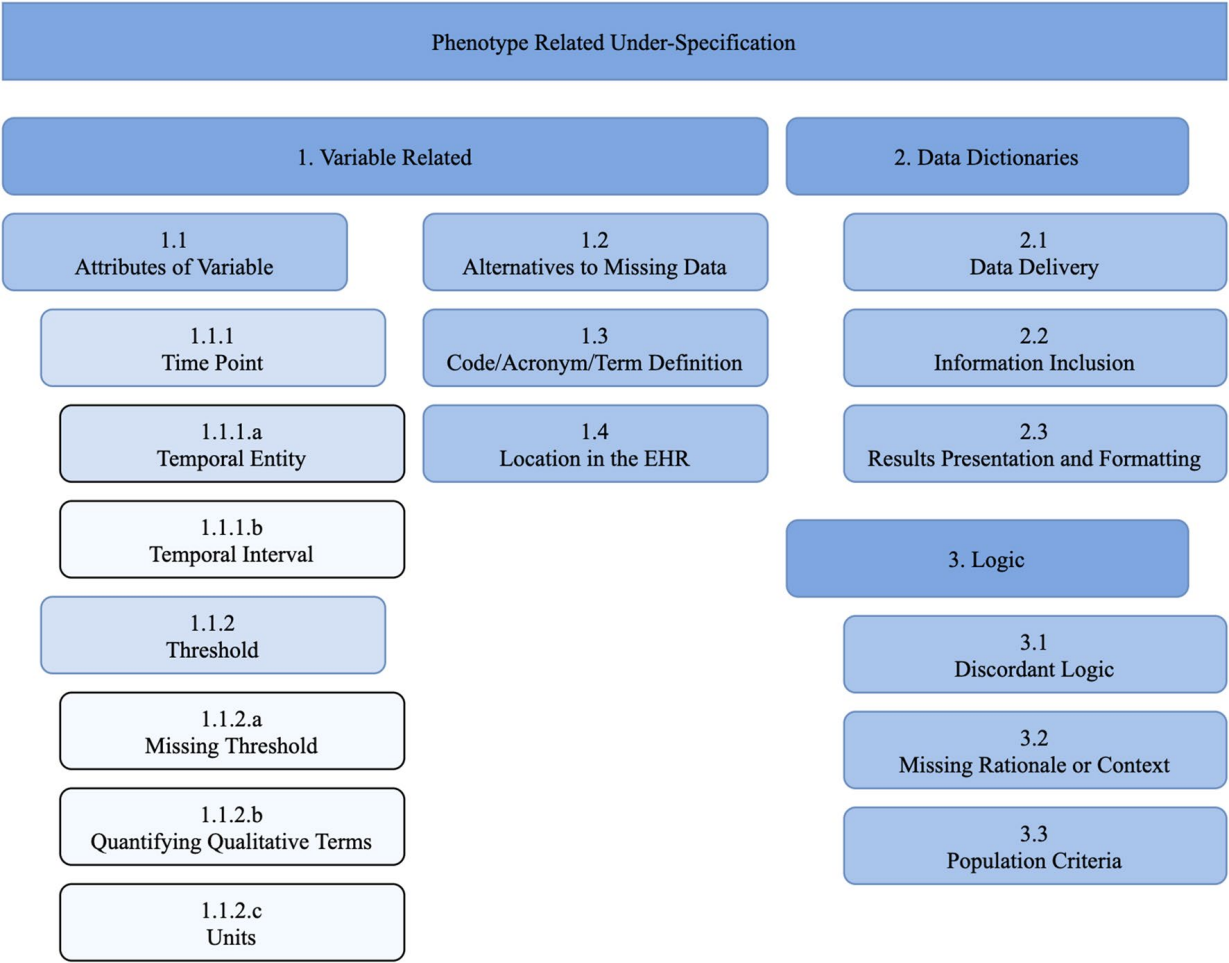
Categories of under-specification and other common issues identified in narrative phenotype algorithms

The final hierarchical taxonomy of under-specifications is presented in Fig. 2 and summarized with descriptions in Table 1. Examples of under-specifications are listed in Table 2. Examples were selected from categories of under-specifications that are present in over 50% of the examined narrative phenotype algorithms. More detailed descriptions of each under-specification category and sub-category are provided in the online supplement.

**Table 2:** Examples of under-specifications in categories with prevalence in over 50% of narrative phenotypes algorithms

Code	Category	Sub-category	Examples
1.1	Definition of variable	Attributes of variable	1. For Bilirubin, do we need to collect total bilirubin, [conjugated], [unconjugated], or all 2. By critical care, do you mean emergency department and/or other "critical" departments, & if so, which types? intensive care, and/or some type of cardiac critical care?
1.1.1.a	Time point	Temporal entity	1. TPN Dx are only excluded if the [sic] occur in the 365 days before first NAFLD Diagnosis code? 2. Which date selected if there are multiple CPTs on multiple dates? What is the definition of the 1st MACE event?
1.3	Definition of variable	Code/acronym/term definition	1. Clarification on use of “3 digit” ICD code 2. Are LOINC codes available for MRSA culture tests?
3.3	Logic	Population criteria	1. Case 1 & 2 criteria are "AND" criteria, i.e., all 3 criteria must be met? 2. How can we define a case who satisfies the criteria defined for both case 1 and 2?
2.2	Data dictionary	Information inclusion	1. Data dictionary indicates that you want height, weight, and BMI as repeated measures. Should the user include all such codes? 2. Do you only want the encounters (LOS) that only have height or weight?

Under-specification categories were mainly based on the common characteristics of a phenotype algorithm such as variables required by the algorithm, data dictionaries for formatting results, and the logic used by the algorithm. Subcategories were created to add an additional layer of detail for identifying and classifying under-specification. For instance, a narrative phenotype algorithm might not have specified the date range required for a cholesterol lab test. This would be coded as an under-specification related to the attributes of a variable, as the date attribute pertains to the variable—cholesterol lab test. It can further be classified as a time point (temporal related) under-specification, which is a subcategory of “attributes of a variable” under-specification.

In the final annotation set, we found varying levels of prevalence for different under-specification categories. The most frequent categories of under-specification were “Attributes of Variable” (n = 47), followed by “Code/Acronym/Term Definition” (n = 28) and “Results Presentation and Formatting” (n = 27). “Attributes of Variable” issues were also found in the greatest number of phenotypes (n = 13, 68%). “Code/Acronym/Term Definition”, “Population Criteria”, and “Temporal Entity” issues were also found in over half of the phenotypes examined.

## Discussion

We identified several broad categories of under-specification observed across a set of 34 narrative phenotype algorithms, and we have presented a taxonomy of these observations. Overall, our findings suggest that while narrative descriptions of phenotype logic are a suitable mechanism for disseminating phenotype definitions, under-specification leads to ambiguity and vagueness, and it occurs often enough to pose an impediment to efficient development and correct implementation of phenotype algorithms.

We note three important considerations. First, ambiguity and vagueness as a result of under-specification were identified in all of the phenotypes reviewed, which were developed across multiple phenotype authors at 9 distinct institutions. This indicates that this is not an isolated issue and that we can expect this phenomenon to be prevalent in other narrative phenotype algorithm definitions.

Second, vague and under-specified phenotype algorithms required additional effort to resolve, thus increasing the overall implementation time for the phenotype algorithm at an institution. Within eMERGE, the use of PheKB served as a central location for the collaborative network to pose questions and allowed subsequent implementing sites to review and learn from the clarifications made (if they were not directly reflected in the phenotype definition). Such requests for clarification are not always made publicly available, such as e-mailing an author directly for clarification, and in these instances each implementing institution may need to request the same clarification. Hence, we can also assume that the issues of under-specification are greater than what was uncovered in this study.

The third consideration is that there may exist instances of ambiguity and vagueness that were not recognized by any implementer. While this is a speculative issue in that our data would not have always uncovered these occurrences, we recognize they can exist and highlight an additional area where misinterpretation may occur. This is particularly risky as it can be subconsciously ignored, particularly with vagueness. An illustrative case is in one of the narrative phenotype algorithms we examined where the original validators of the algorithm missed a case of ambiguity as the algorithm did not specify whether all available BMI values were needed or only values at a specified time point. Other implementers of the algorithm later identified this ambiguity and sought clarification.

The issue of linguistic ambiguities, vagaries, and uncertainty are not specific to the realm of phenotype algorithm development. As phenotype algorithm definitions specify the process for software implementation, we note similar issues identified with requirements specification in the field of software engineering. This includes not only describing ambiguity within software requirement documents, which includes under-specification and vagueness [13–15], but also considerations and tools for automated detection of these linguistic constructs [16, 17]. Requirement specifications are not directly equivalent to phenotype definitions; requirements typically describe the objectives of what should be built, whereas the phenotype is more a representation of what has been built and should be replicated. However, similarities in detection of under-specification may be applied and warrant further investigation.

Within the healthcare domain, the use of “hedge terms” (intentional expressions of uncertainty) within clinical notes has been reported, including a review of the literature identifying 313 hedge phrases, and an analysis revealing the 30 most prevalent hedge phrases used in a clinical note corpus [18]. These are artifacts of the uncertainty of medicine and the diagnostic process, which could be simple phrases such as “possible”, “likely”, and “unlikely” or more complex group concepts such as “clinically significant infection”, which require further specification using contextual knowledge. Hedge terms typically represent a different source of vagueness that, although more frequent in documenting the clinical process, could still occur in phenotype algorithm definitions.

Similarly, the classification of ambiguity and vagueness within clinical practice guidelines (CPGs) has illustrated complementary findings that intersect the previously mentioned study on hedge terms in clinical notes, as well as the work described here on phenotype algorithm definitions [9]. In this work, the authors conducted a literature search and developed a 3-axis model to classify CPG ambiguity and under-specification. Axis 1 includes linguistic definitions of ambiguity, vagueness, and under-specification, and aligns with our described model. Axis 2 considers if a vague statement is potentially deliberate, and Axis 3 looks at the affected portion of the CPG—both of which are irrelevant to phenotype algorithms.

Our findings provide insight into the issue of vague and under-specified phenotype definitions, and we believe this heightened awareness can be used to guide phenotype algorithm developers to mitigate its detrimental effect. We propose potential solutions, based on our findings, that would mitigate the risk of vague and under-specified phenotype definitions.

First, we believe that explicit enumeration of categories of under-specification in phenotype algorithms raises awareness of these potential issues amongst phenotype algorithm developers. By becoming familiar with ambiguity and vagueness caused by under-specification, developers can be more mindful when writing future narrative phenotypes algorithms and be more attentive to these issues. In particular, having a list of categorized ambiguities to avoid can serve as a handy checklist when composing and reviewing an algorithm definition.

Second, additional resources are needed (including methods, tools, and standard terminologies) to further assist in reducing ambiguity and vagueness from under-specification. This includes approaches for identification and detection of “red flags” like hedge terms. Once developed, narrative phenotype algorithms could be cross-checked by hand and potentially supplemented by computable means before completion. This allows the developers to identify potential issues prior to validation or implementation. Several categories of under-specification are due to the lack of quantification for qualitative terms, such as not having a numeric threshold for “obese”. A similar check for qualitative descriptors and attributes of variables used by the algorithm would be beneficial for reducing ambiguity and vagueness.

Third, as noted in the software engineering space [16, 17], semi-automated approaches may be an approach to assist phenotype authors in detecting these issues, in addition to provided guidelines. The use of natural language processing (NLP) and natural language understanding (NLU) can process and discern relationships between the entities found in the text. For instance, with “BMI at age 21”, NLU can establish the relationship between BMI and age. Systems such as Criteria2Query have demonstrated great progress in this area and could be further adapted for this purpose in the future [19]. While NLP/NLU is not a panacea, such tools can be designed to assist and train phenotype algorithm developers to have better awareness of under-specification. Again, drawing from the software engineering domain, this could be considered as a “linter”—a tool that aids a developer in identifying both errors as well as potential issues.

Lastly, the potential to introduce ambiguity and vagueness through under-specification is mitigated in part with the use of common data models (CDMs) and harmonized terminologies [7, 20–23]. For example, the eMERGE network has more recently begun transitioning to the Observational Medical Outcomes Partnership (OMOP) CDM. CDMs can facilitate the representation of the phenotype algorithm in a computable format, which increases portability of phenotype algorithms while reducing implementation times as it obviates the need for human interpretation of a narrative [24]. It is important to still consider, as computable phenotypes are often the result of a process like BQM, that issues stemming from under-specification could unintentionally creep into the final definition. For example, within the Clinical Quality Language (CQL), it is possible to constrain a population based on age using an expression like AgeInYears() ≥ 40. This is a convenient shorthand to express the patient’s age in years as of today (which changes each time the definition is run) and evaluate whether that value is ≥ 40 years [25]. That statement is not vague from the standpoint of a system that executes CQL, as there are agreed semantics in the interpretation of this expression. However, the author of the CQL expression may not have considered the implication of this expression, where a more expressive statement such as AgeInYearsAt(Today()) ≥ 40 explicitly describes that the author intended the age to be evaluated in the context of “today” each time the CQL expression is run. Therefore, it is important to ensure computable phenotype definitions are still reviewed.

### Limitations

This study has a few limitations. First, we limited our analysis to the comments posted within one specific research network, which may not mimic the processes used by other phenotype authors or consortia. However, the phenotypes we reviewed represented multiple institutions involved in eMERGE over several years, over which the network adjusted its process based on lessons learned. Second, given the asynchronous nature of the comments, and the potential for external communications to have taken place, the collection of questions and answers we analyzed does not provide an accurate measurement of the effort needed to resolve each case of ambiguity and vagueness. Future work should prospectively account for this to quantify the level of difficulty to resolve each category under-specification. Third, other examples of ambiguity and vagueness may exist in the full phenotype definition, which we did not review, or may have been expressed via alternate communications to the phenotype author. Fourth, although these phenotypes were developed at different institutions, they were done as part of a collaborative network where authors were exposed to previous phenotype algorithms. We cannot rule out the possibility that this may have generally informed how future phenotypes were written. Given these factors, we recognize our codebook is likely not comprehensive in that it may not cover every possible case of ambiguity and vagueness. However, we believe the analyzed set is a reasonably representative sample, given the number of phenotype authors and diseases covered across all of the phenotypes in this study. Finally, we describe recommendations (drawing from the literature where possible) but have not formally evaluated the impact of these recommendations prospectively. We believe that the identification of this taxonomy is beneficial, and hope that it will support future work to develop and evaluate tools and methods for phenotype developers.

## Conclusion

Ambiguity and vagueness resulting from under-specification was found to be common in all narrative phenotype algorithms we reviewed, regardless of the developer. In practice these issues slow down implementation of phenotypes at multiple institutions and may also impact the accuracy and consistency of phenotype algorithms, especially if they go unnoticed by the implementers. Our study thoroughly examines the characteristics of under-specification within the phenotypes and proposes a taxonomy that defines the categories of under-specification, with the hope of raising awareness of approaches to remediating them.

## Supplementary Information

**Additional file 1. Supplemental Table 1.** Phenotypes from the Phenotype KnowledgeBase (PheKB) that were reviewed.

## Data Availability

The datasets generated and analyzed are not publicly available because they are proprietary to PheKB, the narrative phenotype algorithm database examined in this study. Data is available from the corresponding author upon reasonable request, and we can work with PheKB to release the dataset to interested parties.
