# Supplementary material for: Under-specification as the source of ambiguity and vagueness in narrative phenotype algorithm definitions
Source: BMC Med Inform Decis Mak. 2022 Jan 28;22:23. doi: 10.1186/s12911-022-01759-z (PMC8796627; doi:10.1186/s12911-022-01759-z)
Supplement: Supplementary file 1 — Additional file 1. Supplemental Table 1. Phenotypes from the Phenotype KnowledgeBase (PheKB) that were reviewed. [file 12911_2022_1759_MOESM1_ESM.docx]

| Codebook Development Phase | Phenotype | Authoring Institution(s) | Validation Institution(s) | Count of Comments |
| --- | --- | --- | --- | --- |
| Initial | Abdominal Aortic Aneurysm (AAA) | eMERGE Geisinger Group | Marshfield | 7 |
|  | ACE Inhibitor (ACE-I) Induced Cough | eMERGE Vanderbilt Group | Mayo | 8 |
|  | ADHD | eMERGE CHOP Group | CCHMC | 4 |
|  | Appendicitis | eMERGE CCHMC/BCH Group | CHOP | 36 |
|  | Drug Induced Liver Injury (DILI) | eMERGE Mount Sinai Group | Mayo | 2 |
|  | Familial Hypercholesterolemia | eMERGE Mayo Group | Geisinger | 28 |
|  | Heart Failure (HF) with Differentiation between Preserved and Reduced Ejection Fraction | eMERGE Mayo Group | GHRI/UW | 22 |
|  | Herpes Zoster | eMERGE GHC/University of Washington Group | Vanderbilt | 20 |
|  | Peripheral Arterial Disease | eMERGE Mayo Group | Marshfield, Northwestern | 13 |
|  | Rheumatoid Arthritis | eMERGE Mayo Group | Vanderbilt | 3 |
|  | Severe Early Childhood Obesity | eMERGE CCHMC/BCH Group | CHOP | 6 |
|  | Sleep Apnea | Beth Israel Deaconess Medical Center | Columbia | 3 |
|  | Statins and MACE | eMERGE Vanderbilt Group | Marshfield | 2 |
|  | Venous Thromboembolism | eMERGE Mayo Group | Marshfield | 29 |
| Expanded Review | Age-related Macular Degeneration | eMERGE Marshfield Group | Northwestern | 14 |
|  | caMRSA | eMERGE Northwestern Group | GHRI/UW | 16 |
|  | Cardiorespiratory Fitness Algorithm (eMERGE Mayo Network Phenotype) | eMERGE Mayo Group | Geisinger, GHRI | 26 |
|  | Chronic Kidney Disease | eMERGE Columbia Group | Vanderbilt | 14 |
|  | Colorectal Cancer (CRC) | eMERGE GHC/University of Washington Group | Northwestern | 38 |
|  | Contrast Induced Nephropathy | eMERGE Mayo Group | Columbia | 9 |
|  | Diabetes Remission after Roux-en-Y Gastric Bypass | eMERGE Geisinger Group | Northwestern | 5 |
|  | Diabetes/Hypertension-Associated Chronic Kidney Disease | eMERGE Mount Sinai Group | Marshfield, Northwestern | 31 |
|  | Diverticulosis and Diverticulitis | eMERGE Northwestern Group | Vanderbilt | 16 |
|  | Epilepsy/Antiepileptic drug response algorithm | eMERGE CHOP Group | CCHMC | 4 |
|  | Fluomics | eMERGE Northwestern Group | CHOP, Vanderbilt, Cincinnati, Marshfield, Mt. Sinai | 1 |
|  | Gastroesophageal Reflux Disease (GERD) Phenotype Algorithm | eMERGE CHOP Group | Northwestern | 5 |
|  | Intellectual Disability | eMERGE CHOP Group | Cincinnati | 8 |
|  | Major Adverse Cardiac Events while on Clopidogrel | eMERGE Vanderbilt Group | Mayo | 19 |
|  | Metformin Response | eMERGE Mayo Group | NU | 10 |
|  | Migraine | eMERGE CCHMC Group | CHOP | 18 |
|  | Non-alcoholic fatty liver disease (NALFD) & Alcoholic Fatty Liver Disease (ALD) | eMERGE CCHMC Group | CHOP | 88 |
|  | Ovarian/Uterine Cancer (OvUtCa) | eMERGE GHC/University of Washington Group | Mayo | 47 |
|  | PAD-NLP 2017 | eMERGE Mayo Group | Primary 5 | 13 |
|  | Pneumonia- VUMC eMERGE v5.1 | eMERGE Vanderbilt Group | Columbia | 39 |

**Supplemental Table 1.** Phenotypes from the Phenotype KnowledgeBase (PheKB) that were reviewed.
